# Identification of hub genes and key pathways in the emphysema phenotype of COPD

**DOI:** 10.18632/aging.202432

**Published:** 2021-02-01

**Authors:** Qiunan Zuo, Youyu Wang, Deqing Yang, Shujin Guo, Xiaohui Li, Jiajia Dong, Chun Wan, Yongchun Shen, Fuqiang Wen

**Affiliations:** 1 Department of Respiratory and Critical Care Medicine, West China Hospital of Sichuan University and Division of Pulmonary Diseases, State Key Laboratory of Biotherapy of China, Chengdu 610041, China; 2 Respiratory Ward, Department of Geriatrics, Sichuan Provincial People's Hospital, University of Electronic Science and Technology of China, Chengdu 611731, China; 3 Department of Thoracic Surgery, Sichuan Provincial People's Hospital, University of Electronic Science and Technology of China, Chengdu 611731, China

**Keywords:** chronic obstructive pulmonary disease, emphysema, weighted gene co-expression network analysis

## Abstract

Chronic obstructive pulmonary disease (COPD) is a heterogeneous condition associated with high morbidity and mortality. This study aimed to use weighted gene co-expression network analysis (WGCNA) to explore the molecular pathogenesis of the emphysema phenotype of COPD. After obtaining lung mRNA expression profiles from ten patients with the emphysema phenotype of COPD and eight controls, emphysema-associated gene modules were identified with WGCNA. Among 13 distinct modules, the green-yellow and brown modules showed the strongest correlations with emphysema severity and lung function and were thus selected as hub modules. On gene ontology analysis, these two modules were mainly enriched in immune response, B cell receptor (BCR) signaling pathway, extracellular matrix (ECM) organization, and collagen fibril organization. Pathway analysis primarily showed enrichment in BCR signaling pathways, ECM receptor interaction, and NF-κB and TGF-β signaling pathways for the two hub modules. Several genes, including FCRLA, MS4A1, CD19, FKBP10, C1S and HTRA1, among others, were identified as hub genes. Our results shed light on the potential genetic mechanisms underlying the pathogenesis of the emphysema phenotype of COPD. However, further research will be needed to confirm the involvement of the identified genes and to determine their therapeutic relevance.

## INTRODUCTION

Chronic obstructive pulmonary disease (COPD) is a chronic airway inflammatory disorder characterized by high morbidity and mortality, which represents a severe public health problem worldwide [1]. COPD is a heterogeneous disease and is thus increasingly categorized into different phenotypes based on clinical features, frequency of exacerbations, treatment responses, and clinical prognosis. Chronic bronchiolitis and emphysema represent the most notable phenotypes of COPD [2]. Patients with the chronic bronchiolitis phenotype present chronic airway inflammation, airway goblet cell hyperplasia, and airway mucus hypersecretion. By contrast, emphysema, another common phenotype of COPD, is characterized by elastolytic destruction of the alveolar wall and no evidence of obvious fibrosis, has drawn in recent years increased attention in both clinical and basic research [3–5].

Patients with phenotypes of emphysema show also some distinct clinical features and treatment responses. The former includes worse pulmonary function, lower body mass index (BMI), and more severe of dyspnea than bronchiolitis patients [6]. A study analyzing 342 hospitalized patients with COPD for the first time of acute exacerbation showed that those with emphysema presented a more severe condition and a worse clinical situation than bronchiolitis patients [7]. As a result of these different mechanisms and clinical characteristics, the pharmacotherapy for each COPD phenotype is different. COPD patients with bronchiolitis phenotype are generally administered inhaled corticosteroids (ICS), while results from a prospective study revealed that COPD patients with emphysema phenotype show less improvement in lung function and dyspnea on ICS and long-acting beta-agonist combination intervention [8]. The pathogenesis of the emphysema phenotype of COPD is highly complex, and significant gaps remain about the molecular mechanisms responsible for the different sub-phenotypes. There is thus a clear need to unveil the specific disease mechanisms in order to advance new treatments for these patients.

Transcriptomic studies based on pathological specimens may help find novel insights into the molecular mechanisms of human disease and identify new treatment targets to develop patient-centered treatments. Previous work has profiled gene expression in patients with COPD, but simple analyses of differential expressed genes are not able to obtain full heterogeneity of pathophysiology for COPD, leaving the molecular mechanisms underlying emphysema unclear [9]. Weighted gene co-expression network analysis (WGCNA) is used to identify potential correlations among genes across sequencing or microarray specimens, allowing detection of clusters (modules) of highly correlated genes [9–11]. WGCNA defines gene clusters and reveal relationships among modules and between modules and external clinical traits by using an intramodular hub gene or the module eigengene [9–11]. In this way, WGCNA may be useful to understand molecular mechanisms of disease and to identify reliable biomarkers for more effective diagnosis, prognosis, and treatment. Using new RNA-Seq data, the current study aimed to address the paucity of WGCNA studies related to the characterization of key genes and signaling pathways involved in the pathogenesis of the emphysema phenotype of COPD.

## RESULTS

### Demographic and clinical characteristics of study patients

The current study enrolled 18 patients, with 15 males and 3 females. The mean ages of all patients were 60.6 years. The severity of emphysema was assessed using the Goddard scoring method, based on the HRCT features of each patient (Supplementary File 1). The demographic and clinical data of patients and controls was summarized in Table 1.

**Table 1:** Demographic and clinical information of the study subjects.

**Characteristic**	**COPD (n=10)**	**Non-COPD (n=8)**	***P***
Age (years)	62.4±5.06	58.3±7.9	0.41
Sex (F/M)	1/9	2/6	0.55
Smoking history (packages per year)	41.75±38.33	14.4±12.08	0.19
White blood cell count (/10^9^)	6.55±1.95	4.95±0.88	0.041
Neutrophil count (/10^9^)	4.34±1.91	2.91±0.96	0.076
CAT score	16.7±1.25	9.87±9.35	0.20
FEV1/FVC (%)	52.86±21.28	80.7±3.80	<0.001
FEV1/Pre (%)	57.04±25.34	107.7±17.07	0.002
MMEF (L)	0.70±0.43	2.98±1.10	<0.001
Goddard score	11.1±5.92	0	<0.001

CAT, Chronic obstructive pulmonary disease assessment test; FEV1, Forced expiratory volume in the first second; FVC, Forced vital capacity; MMEF, Maximum mid-expiratory flow. Values are n or mean ± SD, unless otherwise noted.

### Coexpression networks and modules construction

**Figure 1:**
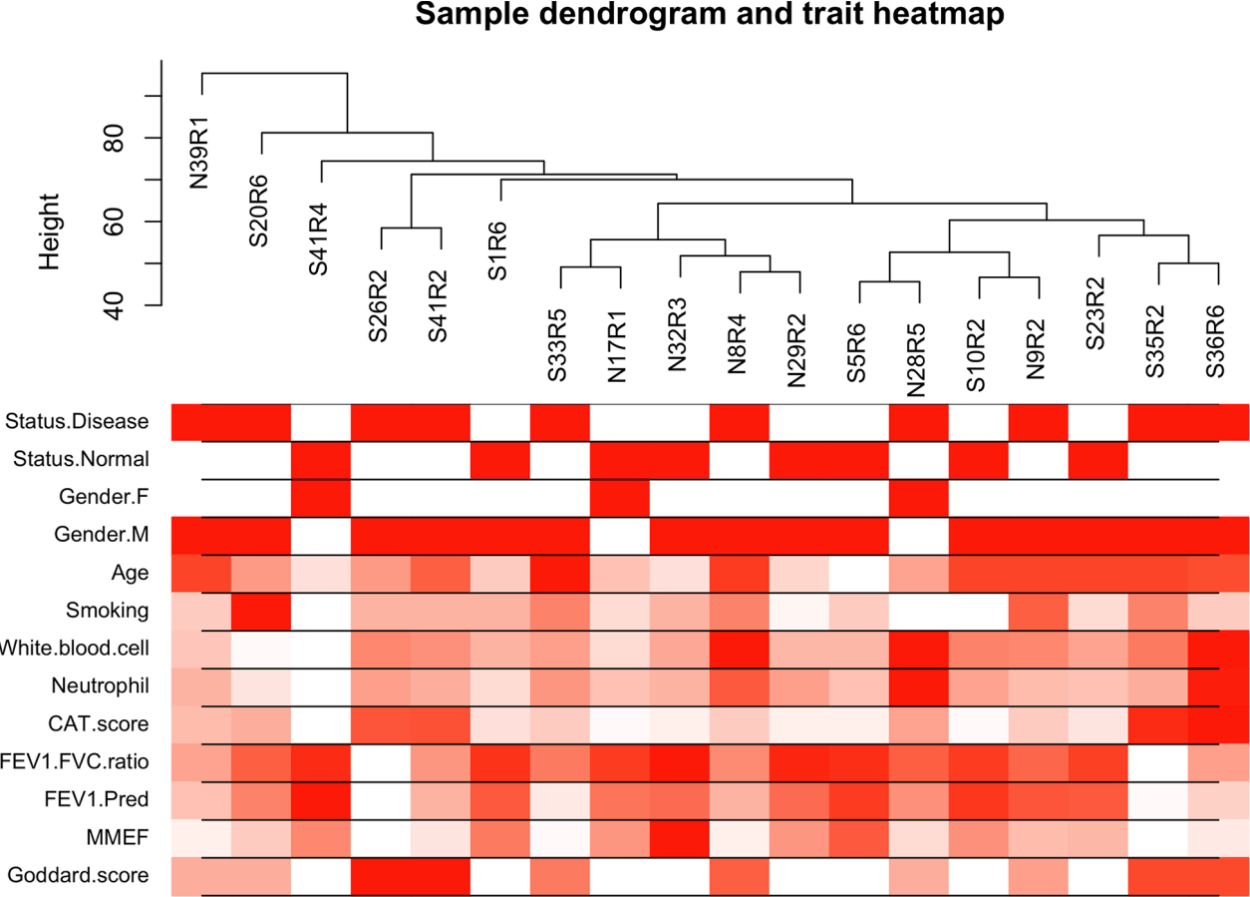
**Cluster tree of clinical samples.** The leaves of the tree correspond to the samples. Color bands represent the numeric values of the physiological traits.

**Figure 2:**
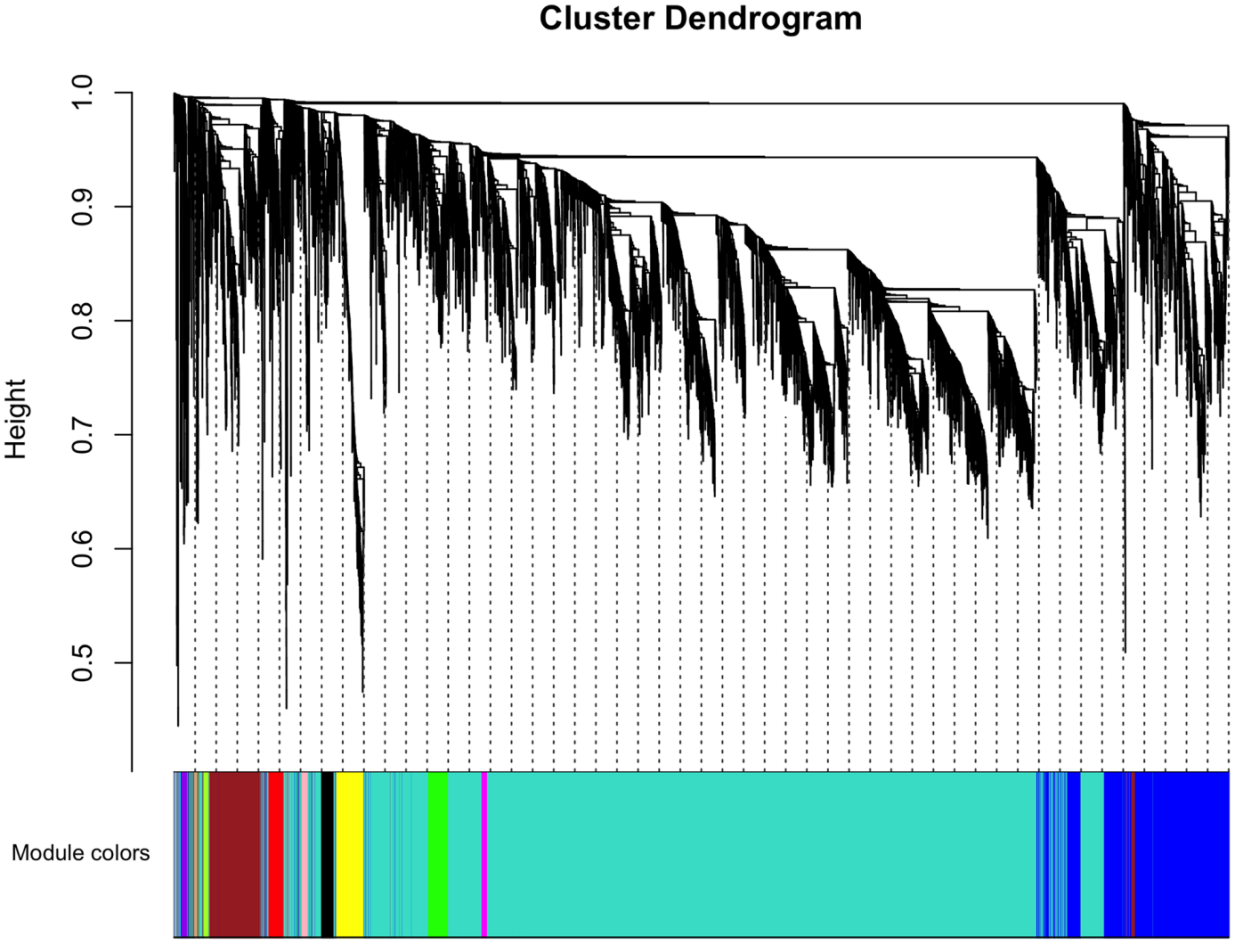
**Cluster dendrogram of gene coexpression and functional modules.** Heatmap plot of the topological overlap matrix (TOM) supplemented by hierarchical clustering dendrograms and module colors. A total of 13 distinct co-expression modules were identified containing tan to turquoise genes. Another 30 uncorrelated genes were assigned to a grey module that was not included in subsequent analyses.

**Figure 3:**
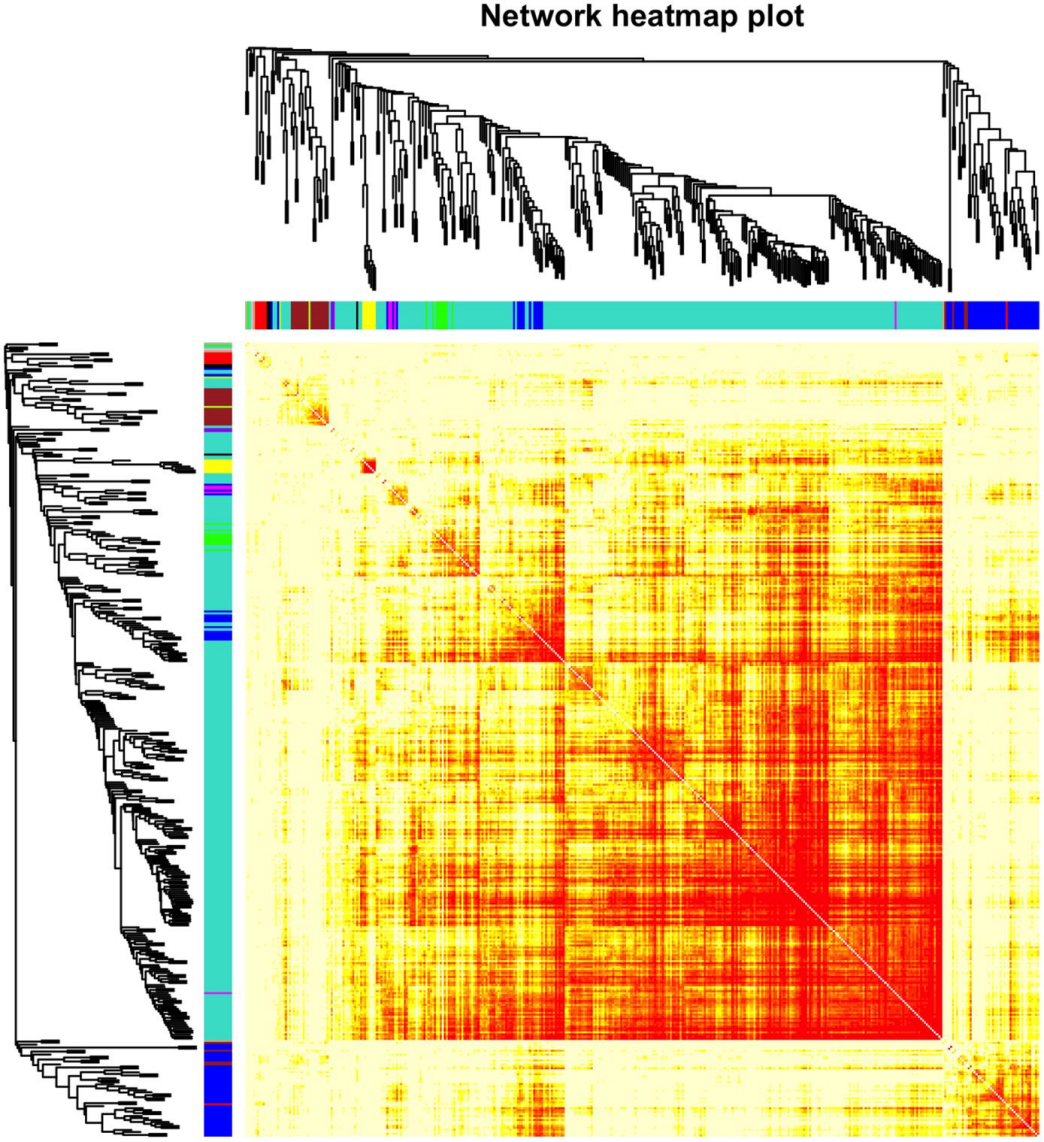
**Heatmap of the gene network.** TOM plot for all the genes in the analysis. Light colors represent low overlap and progressively darker (red) colors represent higher overlap. Darker blocks along the diagonal correspond to modules. The gene dendrogram and module assignment are also shown along the left and top sides.

The top 50% most varying genes (n = 9,550) were used for WGCNA network construction. Thirteen types of clinical data, including disease status (COPD vs. control), sex, age, history of smoking, white blood cell count, neutrophil count, smoking (packages per years), FEV_1_/FVC, FEV_1_/Pre, maximum mid-expiratory flow (MMEF), CAT score, and Goddard score were evaluated. Genes were divided into modules through hierarchical average linkage clustering if they exhibited similar patterns of expression (Figure 1). To assure relatively balanced mean connectivity and scale independence, the network topology was constructed with various soft threshold powers in order and a soft threshold power of 9 was finally chosen to analyze network topology. Using a dynamic tree-cutting algorithm and 0.25 as the merging threshold function, 13 modules were identified in the dataset (Figure 2). A topological overlap matrix (TOM) plot of the gene network was constructed (Figure 3).

### Relationships between clinical traits and modules

**Figure 4:**
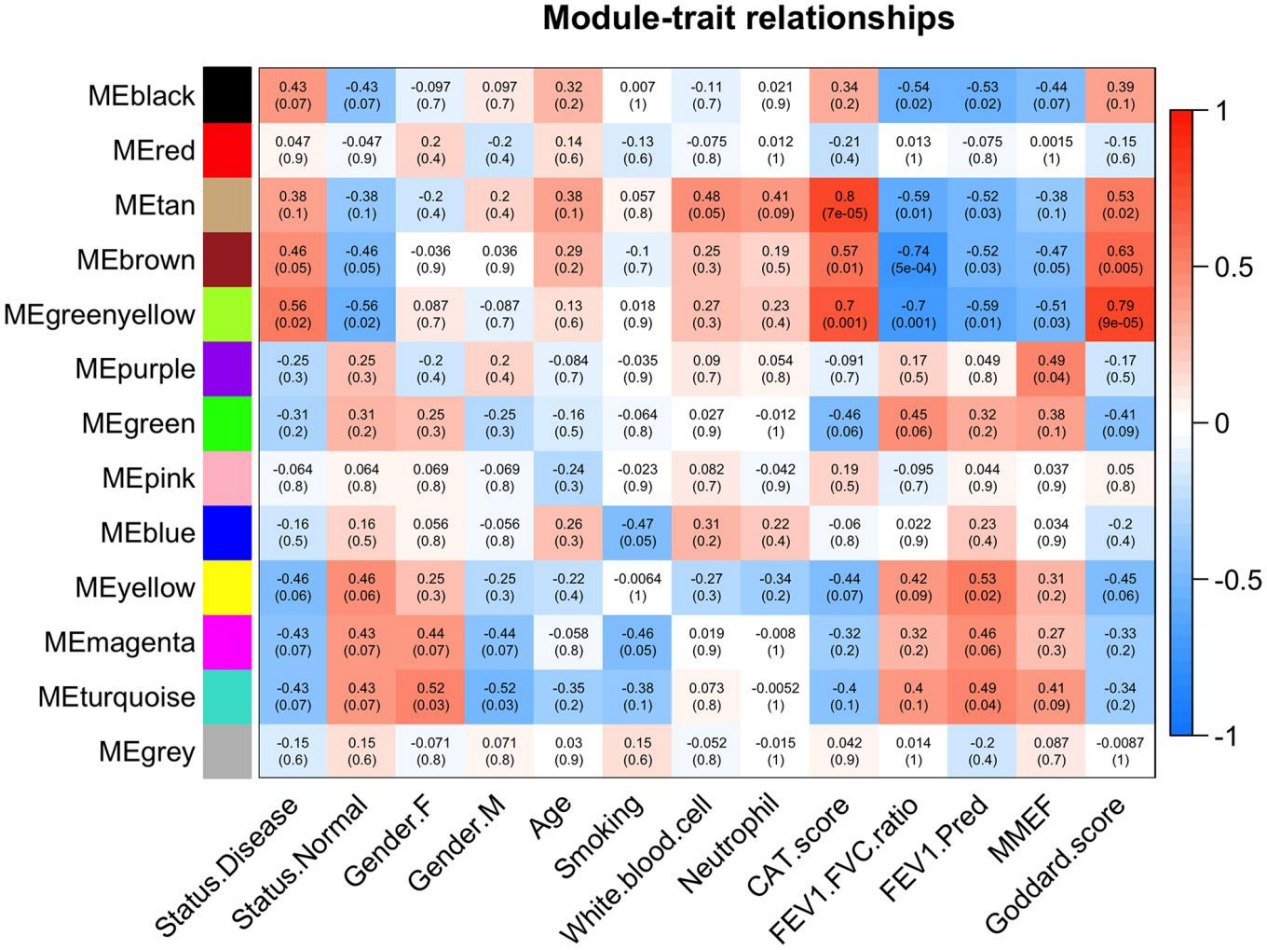
**Module-trait associations.** Each row corresponds to a module eigengene and each column to a trait. Each cell contains the corresponding correlation and p values. The table is color-coded based on correlation values.

Next, the correlations between the 13 module eigengenes (MEs) and traits of interest were evaluated (Figure 4). The brown module was negatively associated with FEV_1_/FVC (r=-0.74, p=5e-04), FEV_1_/Pre (r=-0.52, p=0.03), positively associated with Goddard score (r=0.63, p=0.005), CAT score (r=0.57, p=0.01), and COPD status (r=0.46, p=0.05). The green-yellow module was negatively associated with FEV_1_/FVC (r=-0.70, p=0.001), FEV_1_/Pre (r=-0.59, p=0.01), positively associated with Goddard score (r=0.79, p=9e-05), CAT score (r=0.70, p=0.001), and COPD status (r=0.56, p=0.02). We focused on the relationship between emphysema severity (Goddard score), severity of lung function impairment, CAT score traits, and MEs, which led us to select the brown and green-yellow modules for next analysis.

### Functional annotation of genes in highly correlated modules

**Figure 5:**
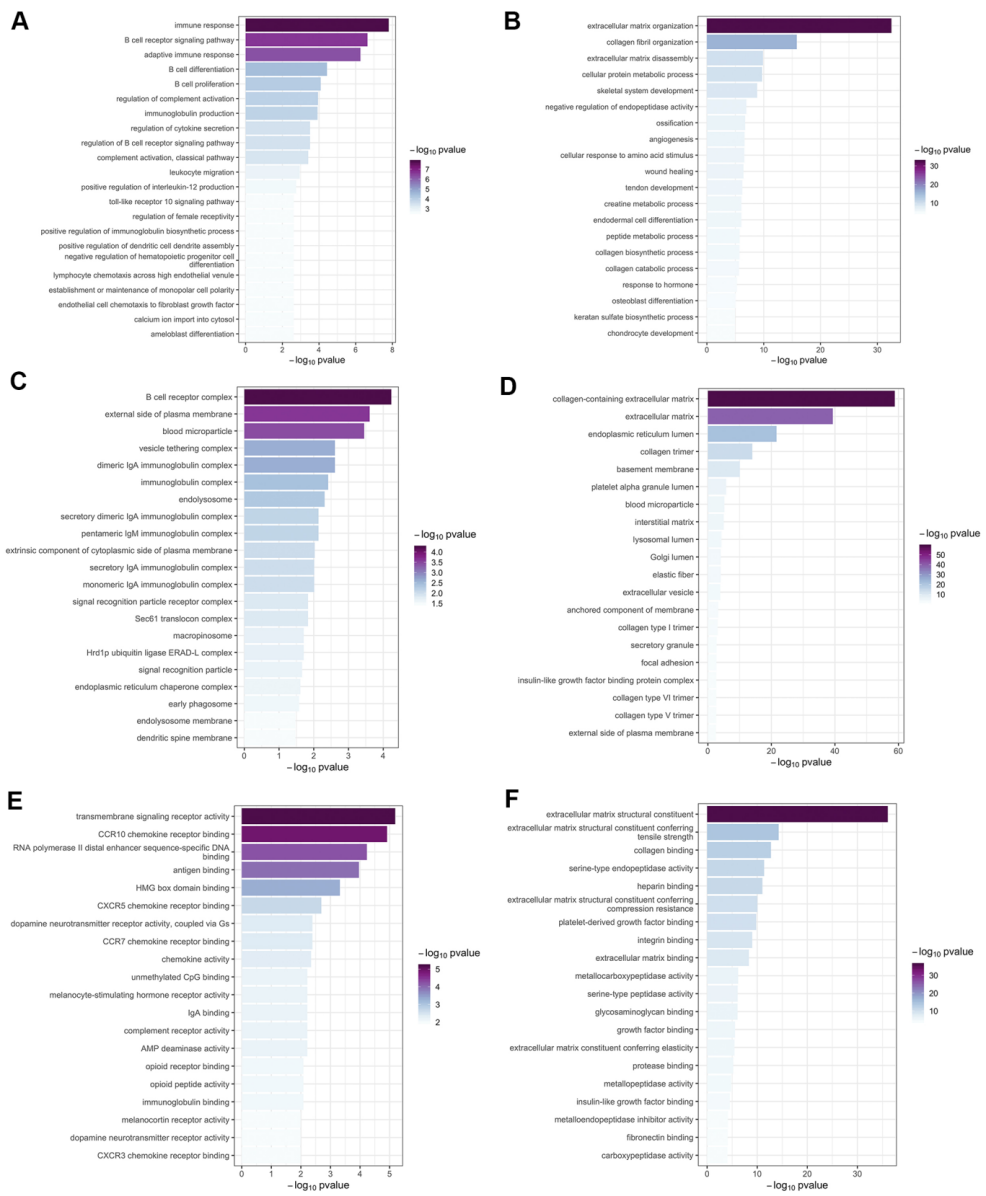
**Gene ontology enrichment analysis.** The top 20 significant (p < 0.05) GO terms were screened in the green-yellow and brown modules. Column color was used to map the p value of specific functional items: darker colors indicate smaller p values (greater significance) for the corresponding enrichment. Results are shown for (**A**) biological process in the yellow-green module; (**B**) biological process in the brown module; (**C**) cellular component in the yellow-green module; (**D**) cellular component in the brown module; (**E**) molecular function in the yellow-green module; and (**F**) molecular function in the brown module.

**Figure 6:**
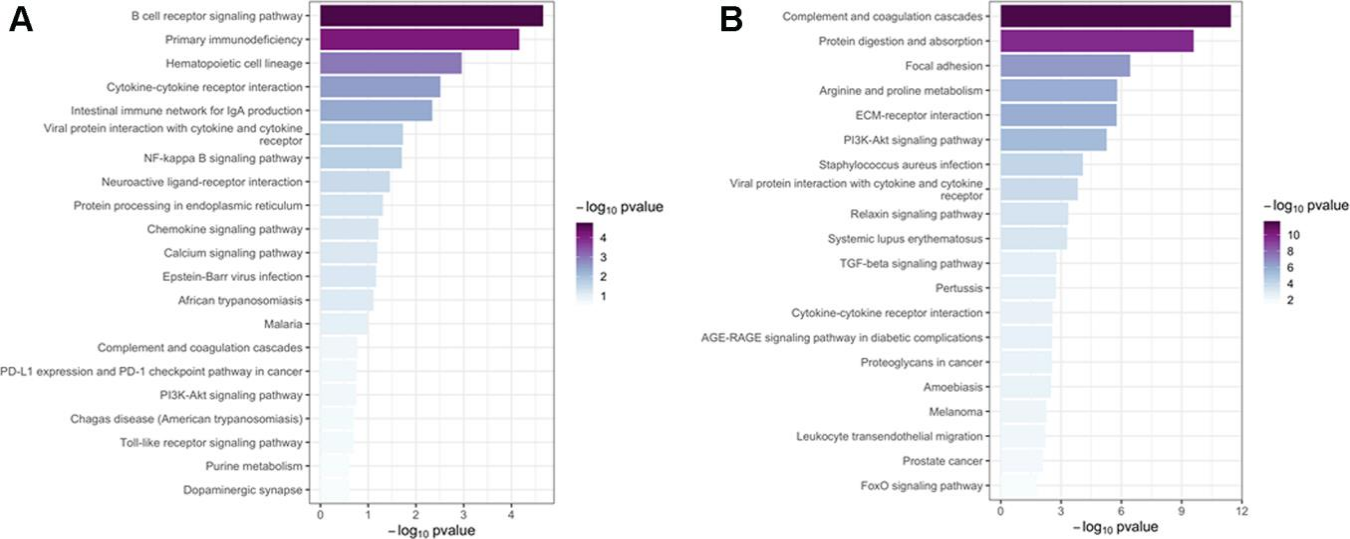
**Kyoto Encyclopedia of Genes and Genomes pathway analysis.** The top 20 significant (p < 0.05) KEGG signaling pathways were screened in the green-yellow (**A**) and brown (**B**) modules. Column color was used to map the p value of specific functional items: darker columns indicate smaller p values (greater significance) for the corresponding enrichment.

There were 47 genes in green-yellow module and 1,402 genes in brown module, respectively. The main biological processes of genes in green-yellow module contained were immune response, adaptive immune response, B cell receptor (BCR) signaling pathway, and primary immunodeficiency; Kyoto Encyclopedia of Genes and Genomes (KEGG) analysis found that the main enriched signaling pathways were BCR signaling pathway, cytokine-cytokine receptor interaction, and NF-κB activity. The genes in the brown module were mainly enriched in the biological processes of extracellular matrix (ECM) organization, ECM disassembly, collagen fibril organization, signaling pathways of the protein digestion and absorption, complement and coagulation cascades, focal adhesion, and TGF-β signaling pathways. The Gene Ontology (GO) terms for biological processes, cellular component, molecular function, as well as the KEGG signaling pathways enriched in genes in the two modules are shown in Figures 5, 6.

### Identification and visualization of intramodular hub genes

**Figure 7:**
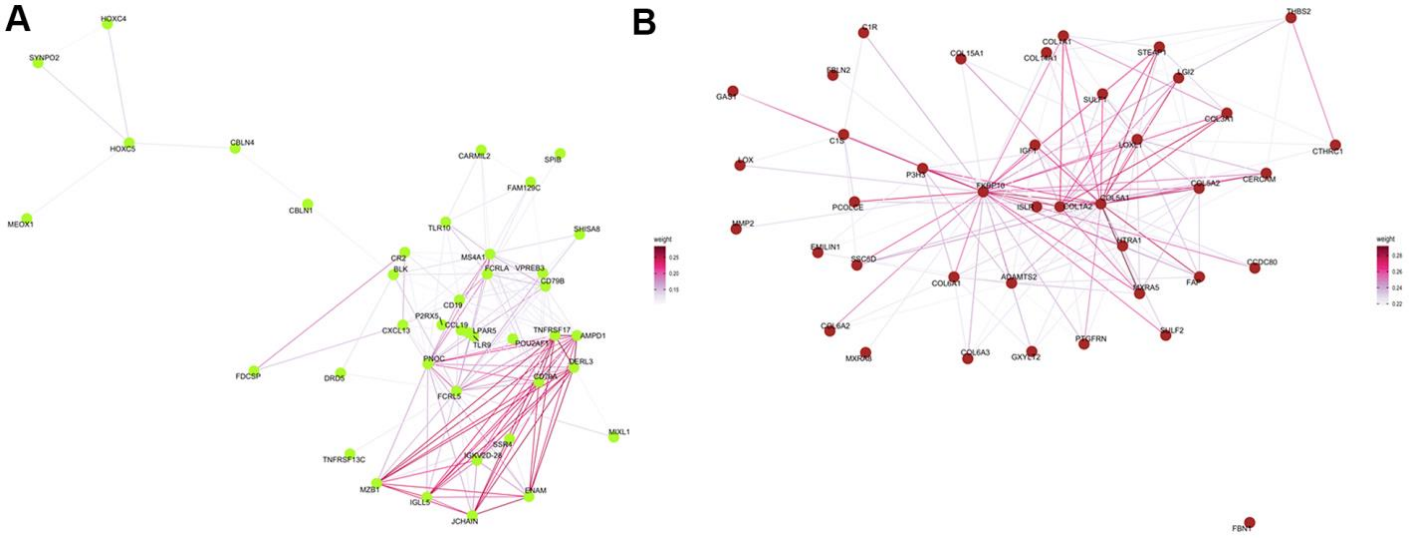
**Protein–protein interaction network analysis.** Visualization of the network connections among the most highly connected genes within the green-yellow (**A**) and brown (**B**) modules. Edge weights represent similarity between nodes.

The Cytoscape was used to visualize the top 40 gene connections and to find the hub genes in each module. The top ten hub genes in the green-yellow module were FCRLA, MS4A1, CD19, FCRL5, CD79A, CD79B, PNOC, BLK, POU2AF1, and VPREB3 (Figure 7A). Genes of FKBP10, C1S, HTRA1, ADAMTS2, EMILIN1, LOXL1, COL6A1, PCOLCE, COL5A1, and COL1A2 were the top ten hub genes in the brown module (Figure 7B). In turn, differential expression analysis indicated that the expression of CD79A, VPREB3, LOXL1, BLK, FKBP10, FCRL5, COL1A2, HTRA1, and CD19 was significantly increased in patients with COPD, relative to controls (all P<0.05, Supplementary Figure 1).

## DISCUSSION

In this study, we used WGCNA to analyze the lung transcriptome of ten patients with emphysema phenotype of COPD and eight controls without COPD. We identified a series of significant pathways, including BCR signaling pathway, ECM-related pathways, NF-κB and TGF-β signaling pathways, and several hub genes (i.e. CD19, BLK, MS4A1, POU2AF1, and COL6A1) that may be involved in the emphysema phenotype of COPD.

In our previous WGCNA study using GSE69818 dataset, IFT88, Bik, MMP10, and CCDC103 were identified as hub genes and were consistently expressed in patients with emphysema, the emphysema related genes were mainly enriched for apoptotic mitochondrial changes, proteolysis, and functions of cilium assembly and movement [12]. However, there was insufficient patient data to comprehensively assess correlations between WGCNA gene modules and clinical characteristics in that study [12]. By contrast, in the present study we were able to correlate lung transcriptome data, obtained with the latest sequencing platform, with emphysema severity (Goddard score), severity of lung function (FEV_1_/FVC, FEV_1_/Pre), CAT score, and other clinical traits. This comprehensive analysis allowed us to select the most correlated gene modules to obtain new insights on the molecular mechanisms potentially affecting the emphysema phenotype of COPD. Attesting to the validity of our research, the current findings overlap partially with those of Faner et al. [13], who also identified MS4A1, POU2AF1, FCRL5, COL1A2 as potential hub genes of emphysema and indicated also the potential involvement of BCR signaling pathway and ECM organization in its development.

Mounting evidence revealed a prominent role of BCR signaling in the pathogenesis of emphysema and COPD. In blood and lung tissue of patients with COPD, increased B cell products (autoantibodies) and B cell numbers were observed, and the B cell-rich lymphoid follicles has been demonstrated in COPD patients with more severe stages [14–16]. In a previous study, a prominent B cell molecular signature occurs preferentially in emphysema; upregulated genes are enriched in ontologies related to B-cell homing and activation, while the immune coexpression network shows a central core of B cell-related genes, which is absent in bronchiolitis cases [13]. The current study found several B cell–related genes (BLK, CD19, MS4A1, CD79B, CD79A, POU2AF1) that were enriched in the green-yellow module. The BCR signaling pathway was also enriched on KEGG analysis, suggesting that BCR is an attractive research target for the emphysema phenotype of COPD.

CD19 is a characteristic marker of B cells. Cigarette smoking, one most important risk factor for COPD, is associated with percentage increases in CD19+ B cells, as well as activated B cells, among peripheral blood mononuclear cells [17]. MS4A1, also named as CD20, is an activated-glycosylated phosphoprotein that interacts physically with BCR and other membrane proteins and regulates the development and differentiation of B cells into plasma cells [18]. LncRNA COPDA1 can increase the expression of MS4A1, then to increase store-operated calcium entry in human bronchial smooth muscle cells, promoting their proliferation and airway remodeling [19]. POU2AF1 expression correlates with emphysema severity, suggesting a potential contributing role [16]. Zhou et al reported that enhancing expression of POU2AF1 alleviated the suppression of host defense genes by smoking in human airway epithelial cells [20]. However, the mechanism of POU2AF1 in emphysema needs further research. Although functional studies are needed to validate the present findings, our study provides further evidence that B cell-associated genes and pathways are involved in the emphysema phenotype of COPD.

Disturbance of the ECM can lead to lung tissue remodeling (including airway wall fibrosis and emphysema) that affects all lung compartments in COPD. Accordingly, several studies have shown that ECM composition is altered in COPD and emphysema patients [21]. For instance, the disruption and remodeling of collagen and elastic fibers surrounding the alveolar airspaces represents a ubiquitous pathological finding in emphysematous human lung samples [22]. Matrix metalloproteinases (MMPs), including MMP 1, 2, 7, 9, and 12, may contribute to the process of emphysematous lung tissue destruction in COPD, which shifts the balance of ECM synthesis and degradation towards degradation [23]. This phenomenon is consistent with the enrichment observed in current study for genes of the brown module in the ECM GO term. MMP-2 and MMP-9 have already been implicated in the pathogenesis of emphysema [24, 25], so they may be potential therapeutic targets in the emphysema phenotype of COPD.

A previous genome-wide association study identified 234 genes that may be associated with lung function in COPD, and several ECM-related processes, including ECM organization, extracellular structure organization, and elastic fiber formation were enriched from these genes [26]. In our study, COL6A1 and EMILIN1, two genes involved in ECM organization, were also enriched in emphysema, and therefore their function in emphysema and COPD merits further research. Increased deposition of collagen in the small airway walls and changes structures denoted mainly by disorganized collagen fibrils are also critical ECM changes in the development of COPD [27]. Changes in collagen and elastic fibers with loss of alveolar attachments result in loose airway walls that deficit alveolar support, which contribute to increased collapsibility of airway, airflow limitation, and alveolar enlargement. Our WGCNA results identified the hub genes in the brown module (i.e. ADAMTS2 and COL5A1) that were enriched in collagen fibril organization. These results support that altered ECM homeostasis is associated with the pathogenesis of COPD.

NF-κB is another potential therapeutic target for the emphysema phenotype of COPD. NF-κB promotes the production of inflammatory mediators, including tumor necrosis factor-α, interleukin-6, and interleukin-1β, growing evidence suggests that NF-κB plays a crucial role in airway inflammation and remodeling processes in COPD [28]. Overexpressed protein arginine methyltransferase-6 may suppress the ability of cigarette smoke extract to activate NF-κB and induce pro-inflammatory gene expression in a murine emphysema model [29]. NF-κB is also involved in the protective process by which erythromycin attenuates MMP/anti-MMP imbalance in cigarette smoke-induced emphysema [30].

We detected enrichment of TGF-β signaling pathway genes in the brown module. TGF-β is necessary for lung organogenesis and homeostasis, and its dysregulation impacts many respiratory diseases, including COPD [31]. TGF-β1 overexpression has been observed in small airway epithelial cells among smokers and COPD patients, suggesting that activated TGF-β signaling is involved in the pathogenesis of COPD [32]. TGF-β overexpression is associated with early events during the emphysematous process [33], and TGF-β dysregulation due to acetylated Smad7 was shown to contribute to emphysema [34]. Future researches are required to determine the mechanism of TGF-β in the emphysema phenotype of COPD.

Despite impressive progress in COPD treatments, no ‘one-size-fits-all’ pharmacological treatment has been discovered. Instead, COPD treatment is relied on clinical symptoms and severity of disease, and focuses on each patient’s individual pathology [35]. The discovery of key pathways involved in COPD, such as B cell-related inflammation, disturbed ECM changes and collagen fibers, updates our perspective on the pathological mechanisms and identifies potential therapeutic targets for COPD with the emphysema phenotype. Still, the lack of functional characterization of some COPD hub genes, like VPREB3, LOXL1, BLK, FKBP10, FCRL5, and HTRA1, suggest that extensive research needs to be undertaken to arrive at more precise and effective therapies.

The findings of the present study should be explained carefully due to potential limitations. First of all, the sample was relatively small and data on some clinical traits like history of exacerbation were not presented, so we were unable to carry out a more comprehensive WGCNA. Second, the Goddard score is a relatively crude measure of emphysema severity, which may bias our results to a certain extent. Third, since lung tissue specimens are hard to obtain, we included lung samples from only 18 patients. Such limited number of specimens and the heterogeneity among them may have a significant effect on the final results. Additionally, we included three cases of patients with comorbidities of lung cancer; since our gene data showed enrichment in the BCR, ECM, NF-κB, and TNF signaling pathways, which are all related to inflammation and tumor, we can’t exclude the possibility of false positive results due to tumor progression [36–38]. Finally, our study did not include mechanistic studies to verify and deepen the insights obtained from our *in silico* analysis. Future work involving a larger patient sample and strict inclusion criteria should build on our results to better understand and treat the emphysema phenotype of COPD.

In summary, we used WGCNA based on lung transcriptome data to build a coexpression network associated with the emphysema phenotype of COPD. We identified two modules and a series of hub genes in association with the emphysema phenotype, which supply new insights into the underlying molecular mechanisms. The findings of this study should be confirmed *in vitro* and in clinical studies.

## MATERIALS AND METHODS

### Lung tissue collection

Lung tissue specimens were prospectively collected from eighteen patients who required thoracic surgery due to a solitary pulmonary nodule (n=12) or lung volume reduction (n=6). The cases were defined by both high-resolution computed tomography (HRCT) identified emphysema and spirometer-detected COPD with the diagnostic criteria set by Global Initiative for Chronic Obstructive Lung Disease [1]. The study included six cases of lung volume reduction, three cases of pulmonary nodule due to lung cancer, and one case of inflammatory pseudotumor. The controls were patients with benign inflammatory nodules without COPD and signs of emphysema on HRCT. Normal lung tissue under microscopy examination was taken at least 5 cm from the tumor if the patient underwent resection surgery for lung tumor or nodule. The collected lung tissue was immediately stored at -80° C. All samples were histologically analyzed using standard hematoxylin and eosin staining [39, 40].

All participants provided written informed consent for their anonymized clinical data to be collected and published for scientific research purposes. This research was approved by the Ethics Committee of Sichuan Provincial People's Hospital (Number: 2020-94).

### Clinical data collection and emphysema evaluation

Demographic and clinical data including age, sex, history of smoking, white blood cell count, neutrophil count, COPD Assessment Test (CAT) score, and spirometry examination were collected for all subjects on admission. Emphysema was detected by HRCT (Siemens, Germany), which is part of routine clinical management before surgery at our hospital. Technical parameters of HRCT were the following: 1-mm collimation, 1–2 mm slice thickness, 120–140 kV, 75–350 mA, and 0.75–1 s scanning time.

The Goddard score was used to analyze the severity of emphysema based on lung HRCT [41]. The interpretations of chest image were carried out at window levels of 2600-2900 Hounsfield units (HU) and at 800–1500 HU window widths, which are the best conditions for detecting emphysema. Six images in three levels of both lung slices (the aortic arch, carina, and 1-2 cm above the highest hemidiaphragm) were analyzed, and the images were scored as normal (0), 5% affected (0.5), 25% affected (1), 50% affected (2), 75% affected (3), or >75% affected (4). The total score ranged from 0 to 24, and the scores were determined independently by two pulmonologists.

### RNA sequencing

Total RNA in lung tissues was extracted using TRIzolH reagent (Invitrogen, Burlington, ON, Canada), following the manufacturer’s protocols. A Qubit Fluorometer (Thermo Fisher Scientific, Waltham, MA, USA) was chosen to assess the yield and purity of the RNA. The integrity of RNA was examined in a 1% agarose gel with RNA 6000 Nano Assay Kit and an Agilent 2100 Bioanalyzer (Agilent Technologies, Santa Clara, CA, USA). RNA from each specimen (5 μg) was sent to isolate poly(A) mRNA, then a non-directional Illumina RNA-Seq library was created with an mRNA-Seq Sample Prep Kit (Illumina, San Diego, CA, USA). A Bioanalyzer Chip DNA 1000 series II (Agilent Technologies) was used for Library quality control and quantification. Every RNA-Seq library had an insert size of 2 base pairs (bp), and 150-bp sequences were created using an Illumina HiSeq 4000 system.

### WGCNA

First, a weighted correlation network was generated by constructing a matrix of pairwise correlations between all pairs of genes chosen according to variance. Comparability was assessed by correlation analysis of connectivity and average gene expression. Connectivity was assessed with the function of soft connectivity in the software R with WGCNA package [42], which was also used to construct the WGCNA in this study. Cluster analysis using the Flash Cluster package in R was carried out. A module was defined as group of genes that show similar patterns of connection strengths with all the other genes in the network, and these groups of gens often have similar functions. Modules were determined using the cut-tree Hybrid function in the software R with Dynamic Tree Cut package.

To identify MEs, the module eigengenes function in the WGCNA package was chosen for calculation. The signed module membership was used to evaluate the correlations between individual genes and MEs. The correlation was used to assess whether a given gene belonged to a specific module. Correlations between MEs and interested clinical traits were calculated to find gene modules whose expression patterns were associated with specific clinical traits. Gene significance (GS) for individual gene was set as the correlation between its expression level and the presence of a specific clinical trait, using the plot module significance in the WGCNA package. The clinical traits evaluated in our study included: disease status (COPD vs. control), age, sex, history of smoking, white blood cell count, neutrophil count, CAT score, and Goddard score. Functional annotation of all genes in each module was performed with the Cluster Profiler based on GO and KEGG databases. The top 20 most enriched GO and KEGG function terms (ordered by p value) were visualized graphically. Intramodular hub genes were identified as genes with the maximum module membership values in protein-protein interaction (PPI) network analysis. The top 40 gene connections based on topological overlap was visualized using Cytoscape software [43]. The expression levels of top 10 intramodular hub genes were compared between COPD and control using Wilcoxon Rank Sum Test, and a P value < 0.05 was set as significant.

### Statistical analysis

Clinical data of COPD patients and controls are presented as means±standard deviation. Comparisons between COPD and control groups were carried out using nonparametric statistics and chi-squared test. Differences associated with p<0.05 were considered significant.

## Supplementary Material

Supplementary Figure 1

Supplementary File 1
