# Supplementary material for: Identification of hub genes and key pathways in the emphysema phenotype of COPD
Source: Aging (Albany NY). 2021 Feb 1;13(4):5120–35. doi: 10.18632/aging.202432 (PMC7950259; doi:10.18632/aging.202432)
Supplement: Supplementary Figure 1 [file aging-13-202432-s001.pdf]

SUPPLEMENTARY FIGURE

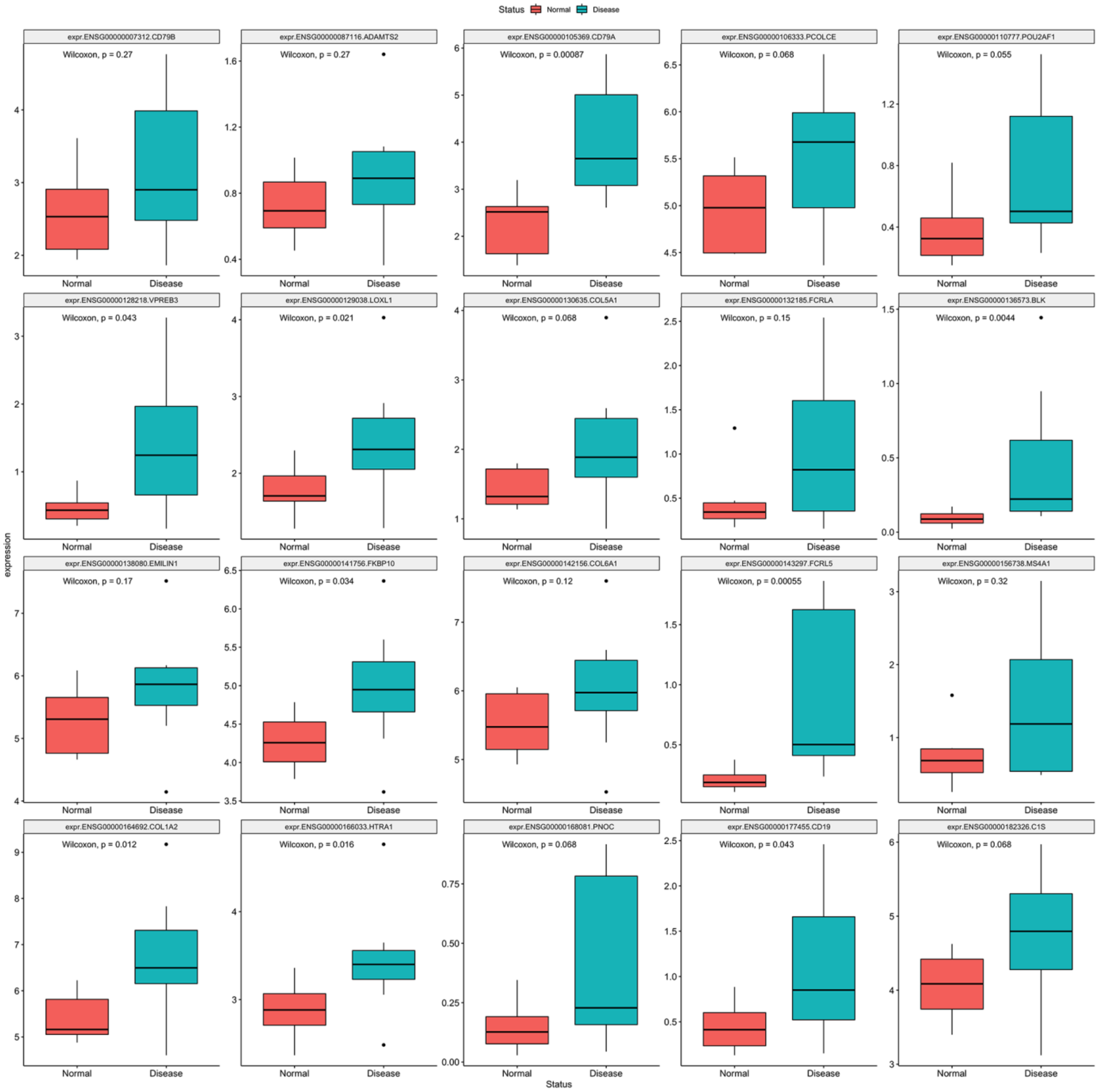

Supplementary Figure 1. Expression of top 10 hub genes in the yellow-green and brown modules.
