# Supplementary material for: Identification of hub genes and key pathways in the emphysema phenotype of COPD
Source: Aging (Albany NY). 2021 Feb 1;13(4):5120–35. doi: 10.18632/aging.202432 (PMC7950259; doi:10.18632/aging.202432)

**Supplementary File 1. CT figure of included patients.**

Methods of Goddard score evaluation: HRCT images were selected at three levels including the aortic arch, carina and 1–2 cm above the highest hemidiaphragm. Six images were analyzed in three slices in the lungs and an average score of all images was considered as a representative value of the severity of emphysema in each person. Each image was classified as normal (score 0), 5% affected (score 0.5), 25% affected (score 1), 50% affected (score 2), 75% affected (score 3) and >75% affected (score 4).

**Patient NO1.** Total score 14=up: 2(R)+4(L) **+** middle:2(R)+4(L) **+** low :1(R)+1(L)


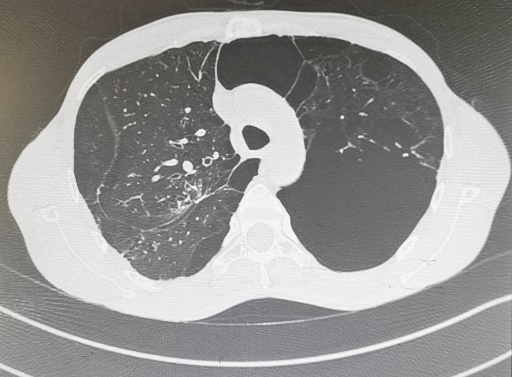

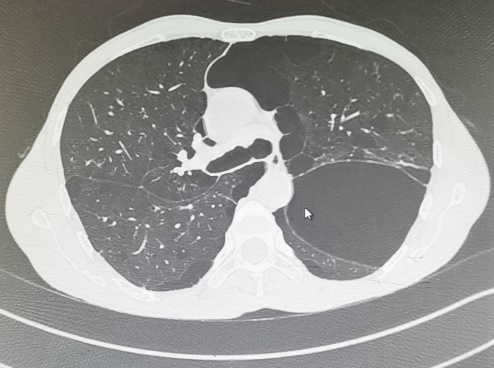

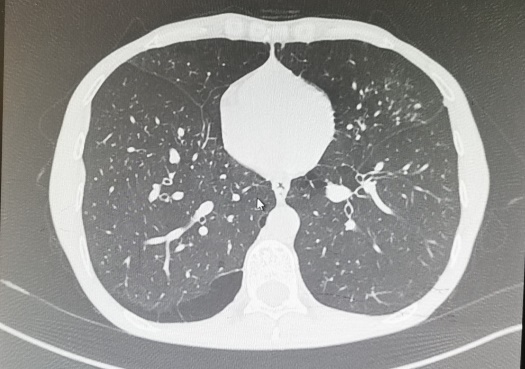


**Patient NO2.** Total score 14= up: 2(R)+2(L) **+**middle:2(R)+2(L) **+** low :4(R)+2(L)


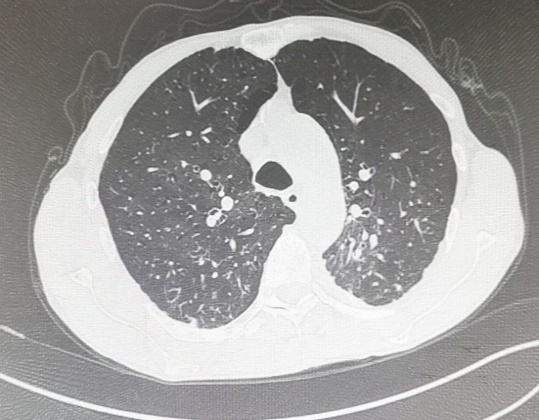

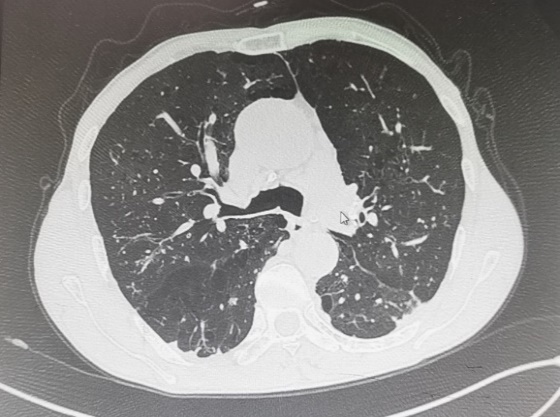

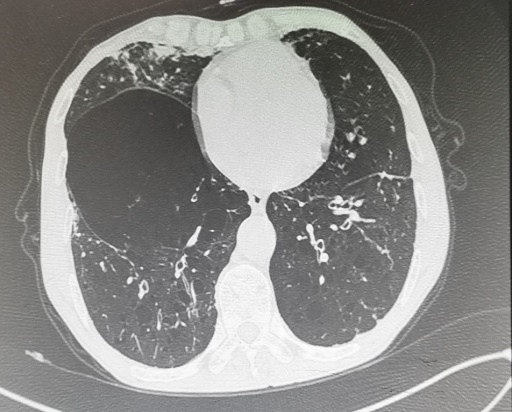


**Patient NO3.** Total score 12= up: 2(R)+2(L) **+** middle:2(R)+2(L) **+** low :2(R)+2(L)


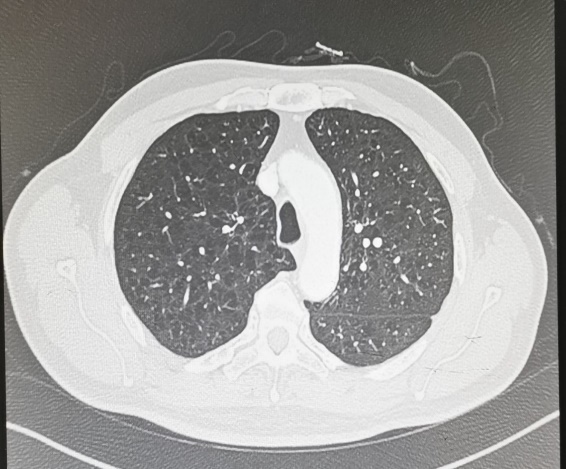

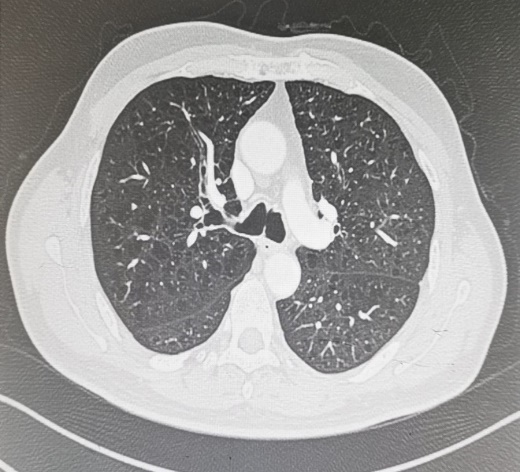

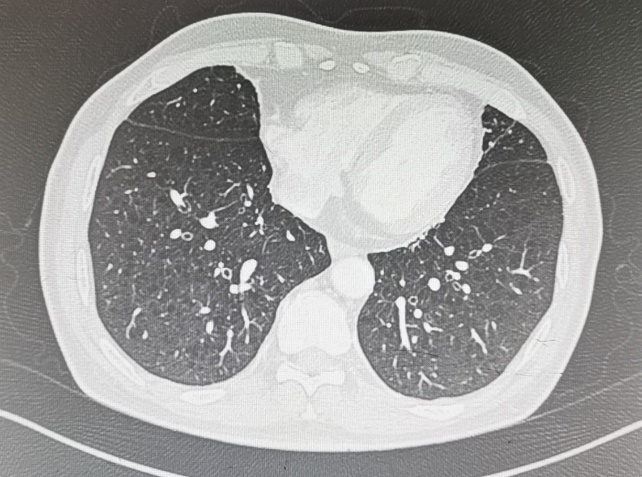


**Patient NO4.** Totalscore 7= up: 2(R)+1(L) **+** middle:1(R)+1(L) **+** low :1(R)+1(L)


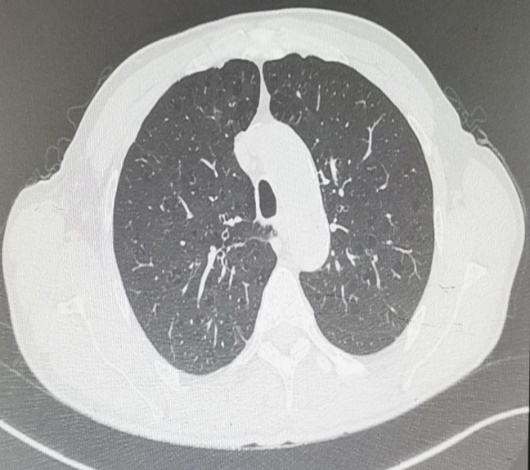

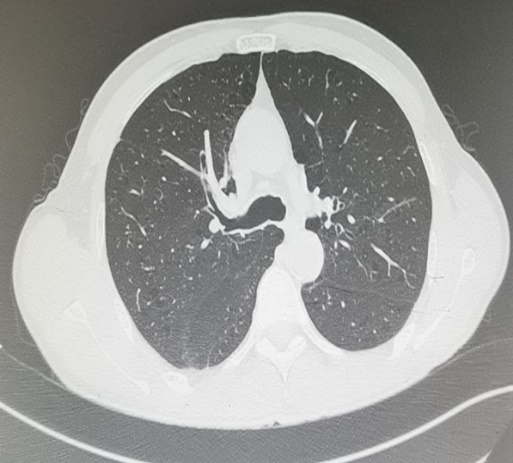

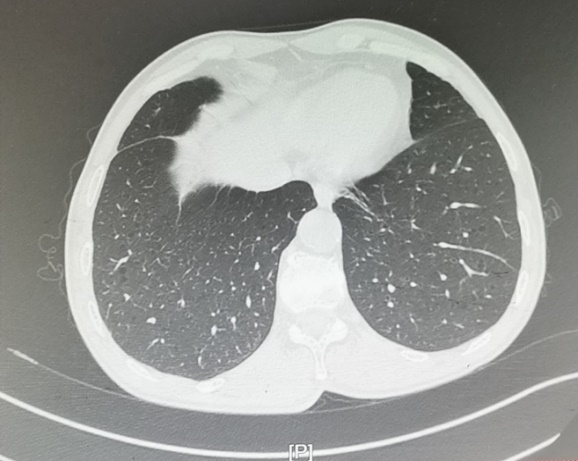


**Patient NO5.**Totalscore12= up: 3(R)+2(L) **+** middle:1(R)+1(L) **+** low :2(R)+1(L)


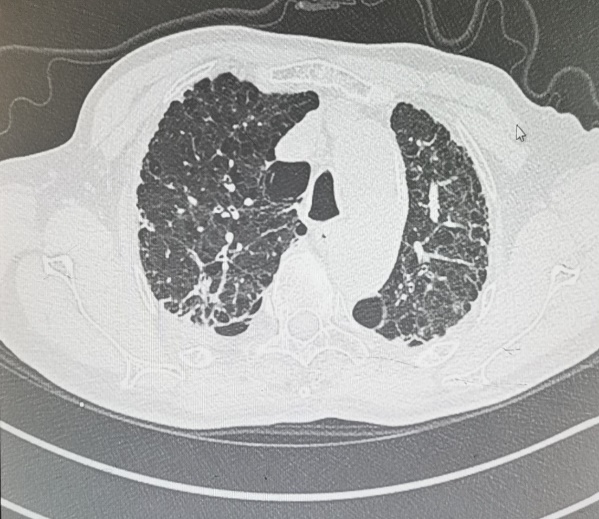

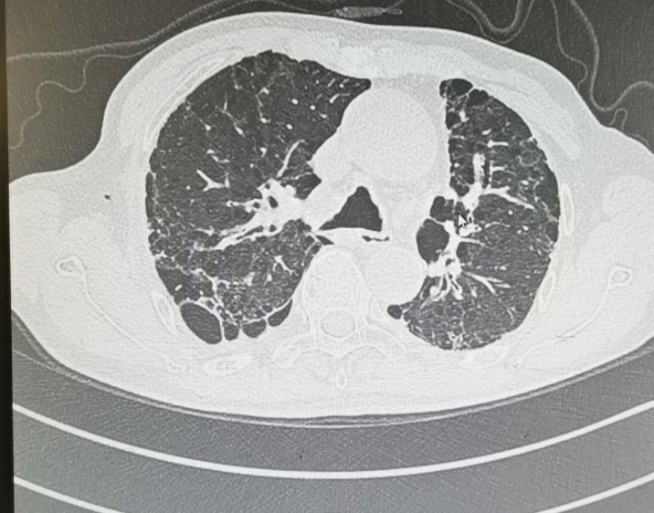

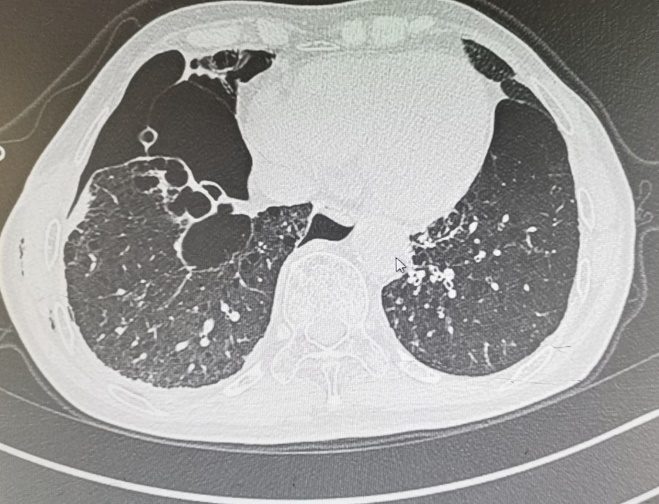


**Patient NO6.** Total score 6= up: 1(R)+1(L) **+**middle:1(R)+1(L) **+** low :1(R)+1(L)


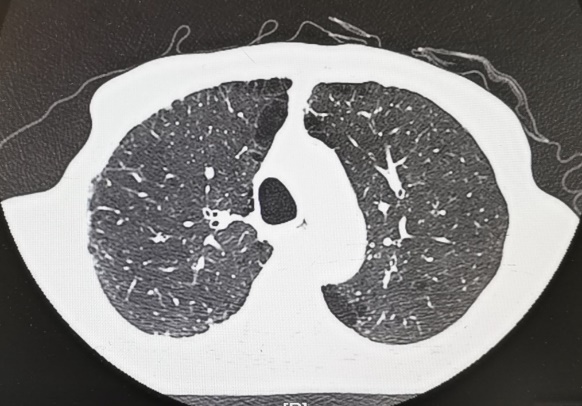

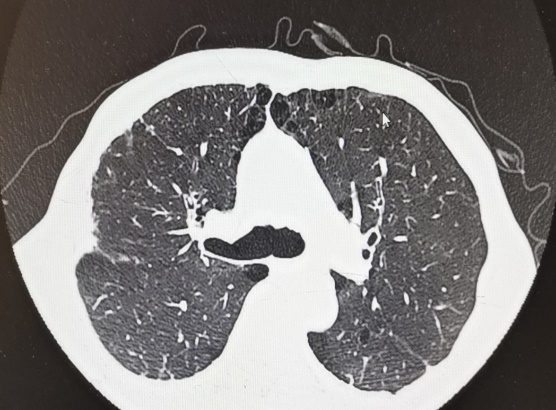

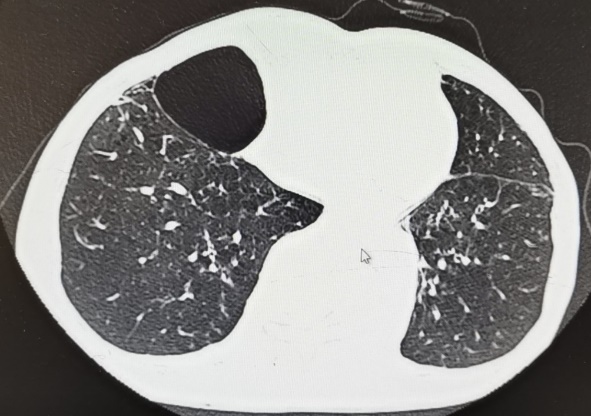


**Patient NO7.** Total score 10= up: 2(R)+2(L) **+** middle:1(R)+2(L) **+** low :1(R)+2(L)


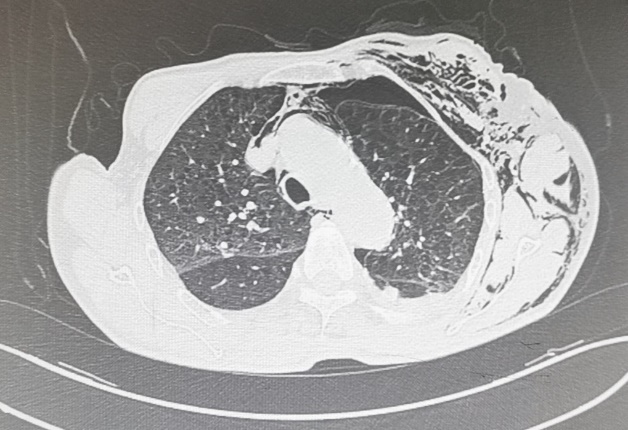

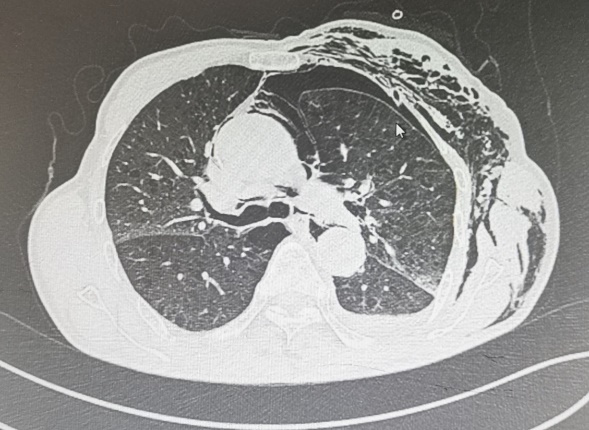

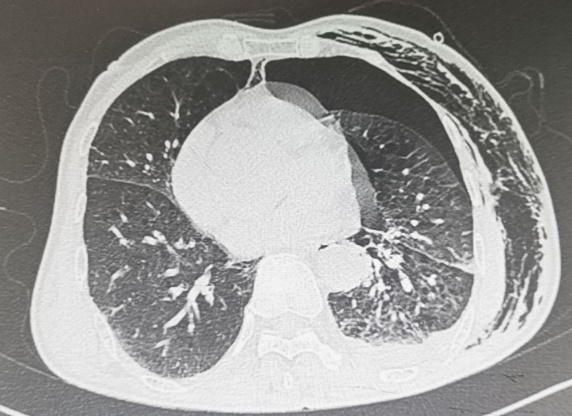


**Patient NO8.** Total score 18= up: 4(R)+2(L) **+** middle:3(R)+3(L) **+** low :3(R)+3(L)


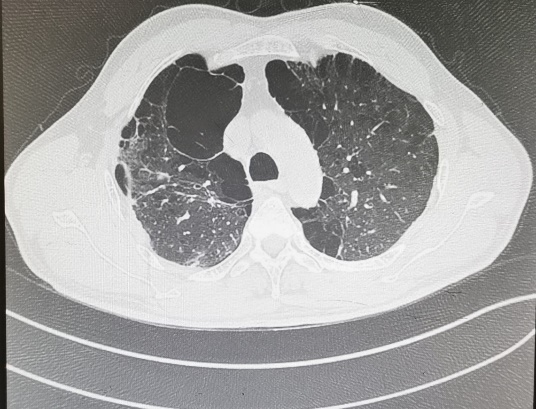

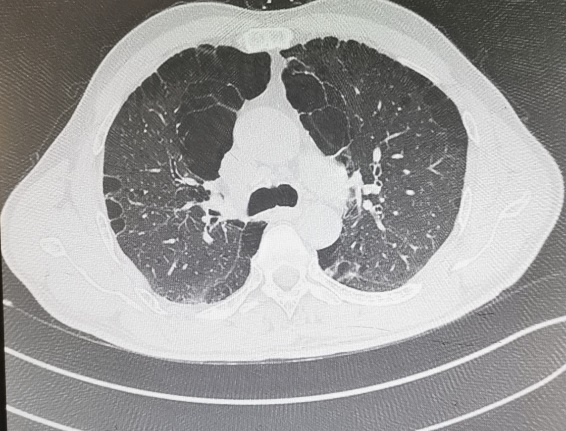

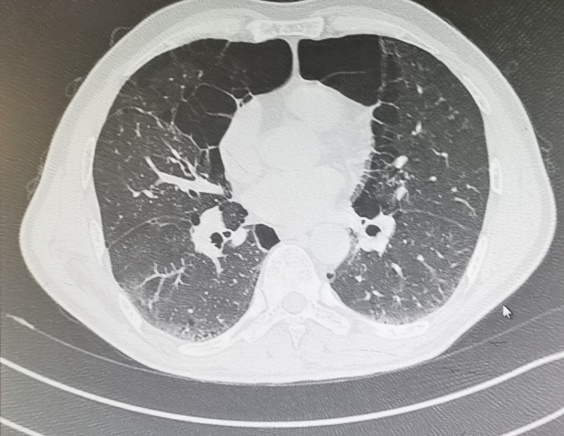


**Patient NO9.** Total score 18= up: 3(R)+3(L) **+** middle:3(R)+3(L) **+**low :3(R)+3(L)


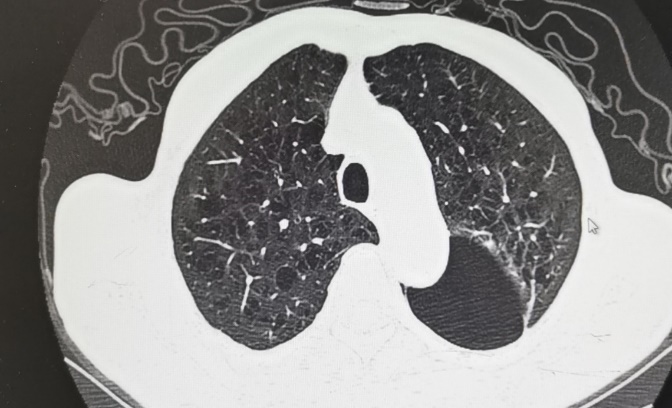

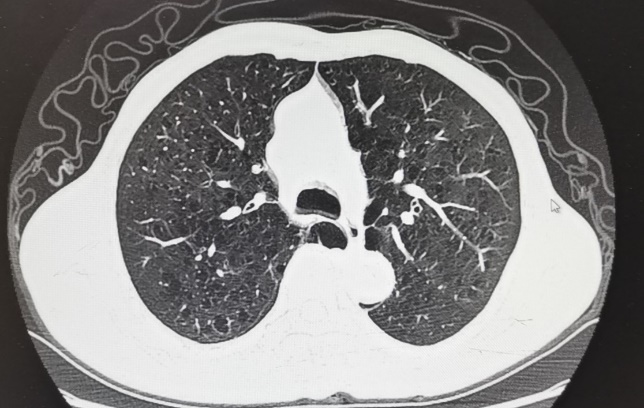

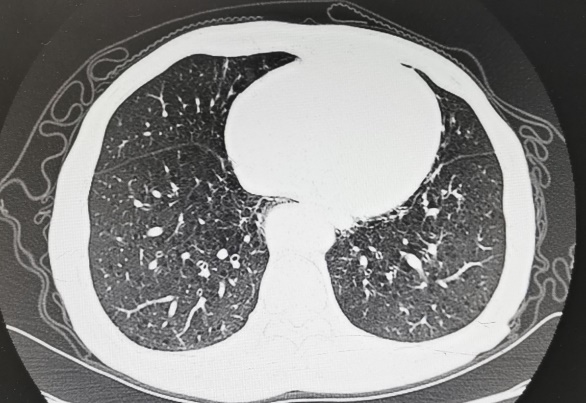


**Patient NO10.** Total score 14 = up: 2(R)+3(L) **+** middle:2(R)+3(L) **+** low :2(R)+2(L)


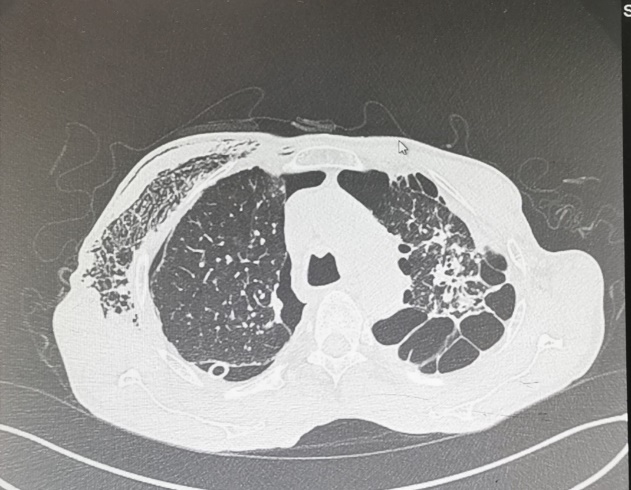

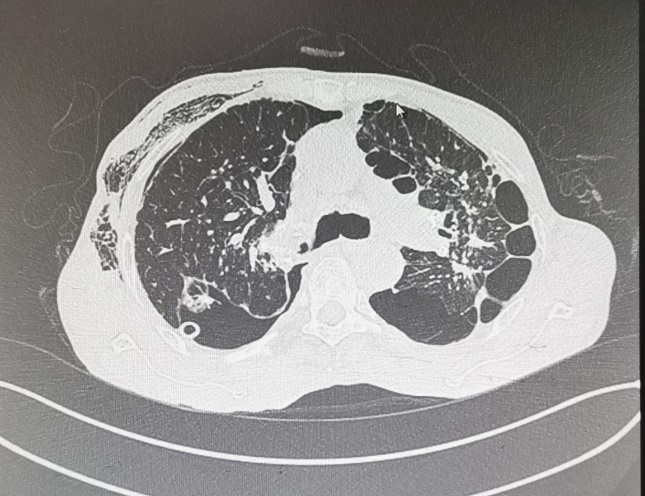

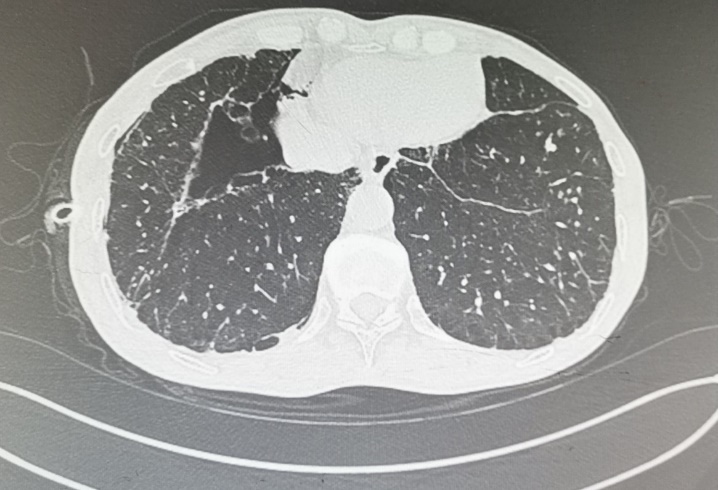


**Controls NO1：**Total score 0 =up: 0(R)+0(L) **+** middle:0 (R)+0(L) **+** low :0(R)+0(L)


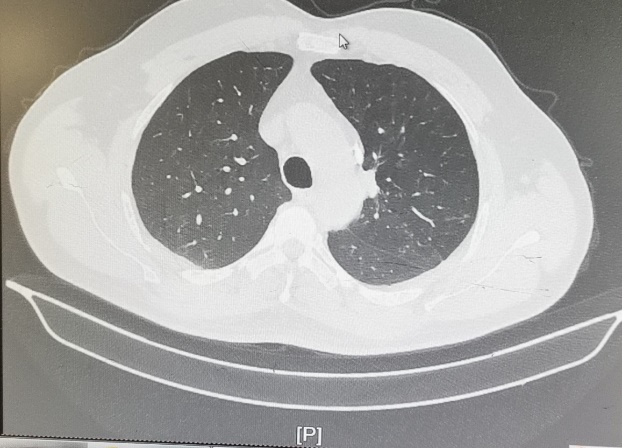

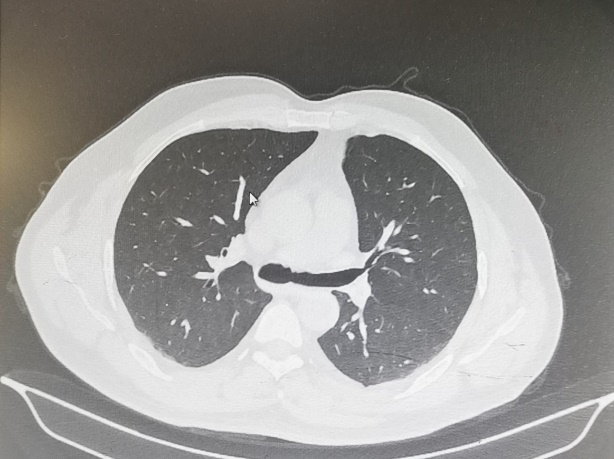

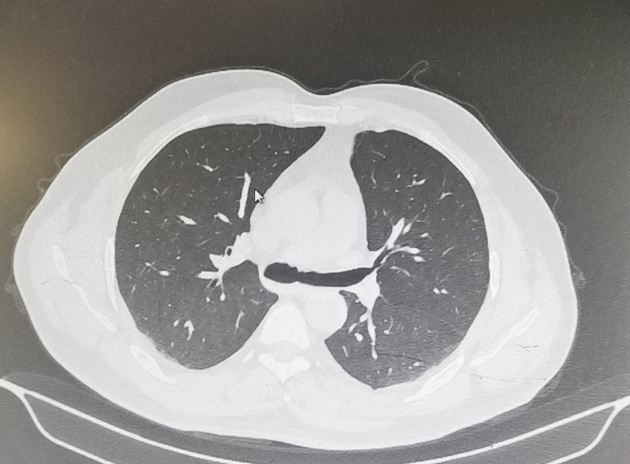


**Controls NO2：**Totalscore 0=up: 0(R)+0(L) **+** middle:0 (R)+0(L) **+** low :0(R)+0(L)


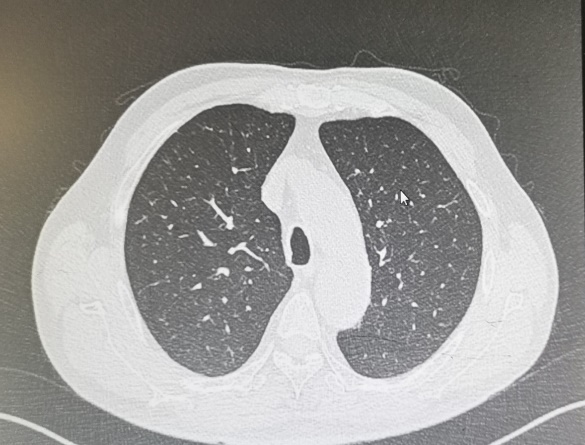

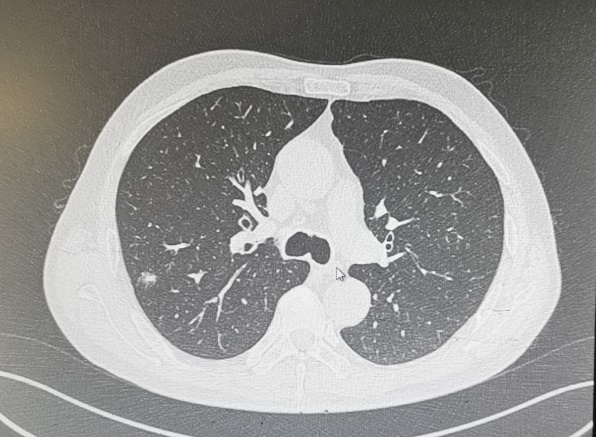

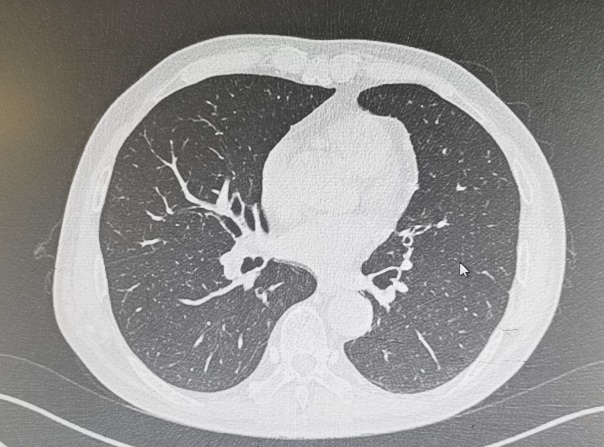


**Controls NO3：**Total score 0=up: 0(R)+0(L) **+** middle:0 (R)+0(L) **+** low :0(R)+0(L)


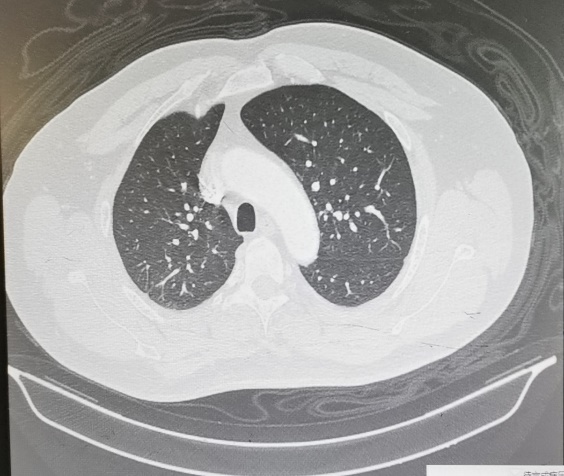

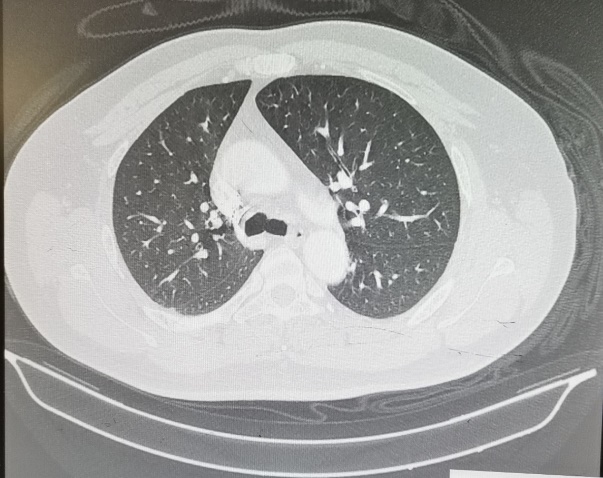

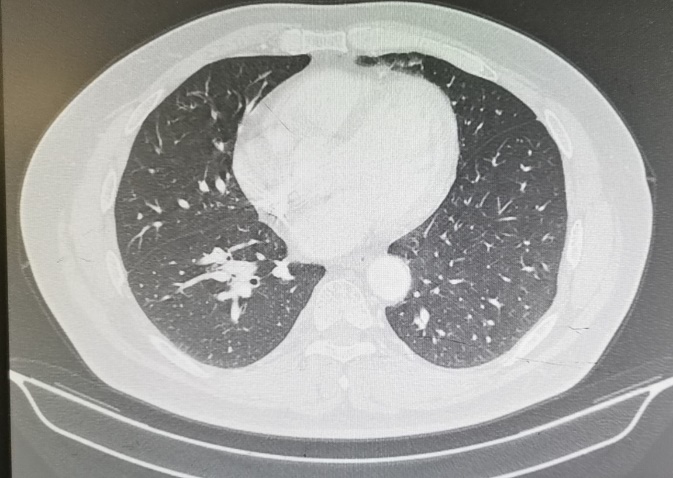


**Controls NO4：**Total score 0=up: 0(R)+0(L) **+** middle:0 (R)+0(L) **+** low :0(R)+0(L)


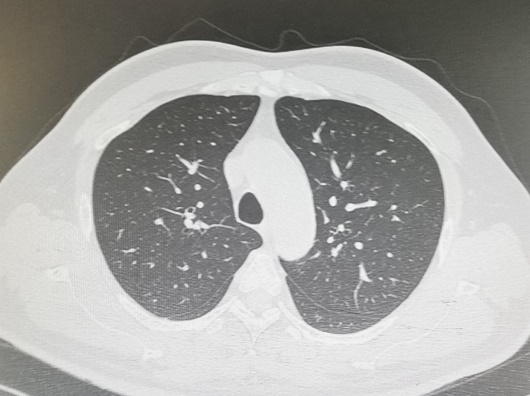

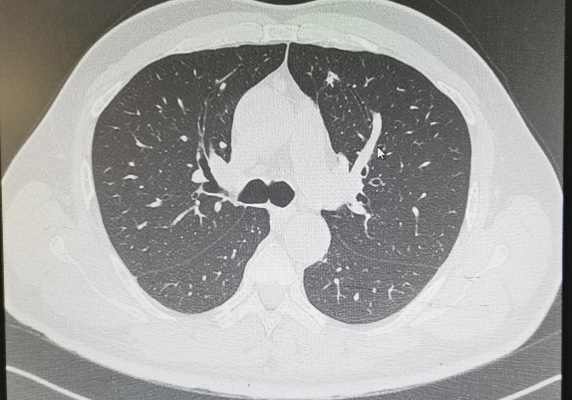

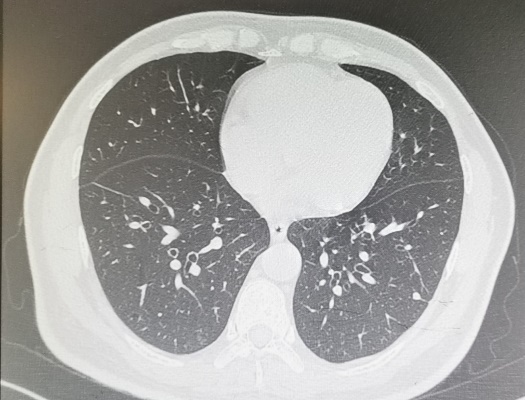


**Controls NO5：**Total score 0 =up: 0(R)+0(L) **+** middle:0 (R)+0(L) **+** low :0(R)+0(L)


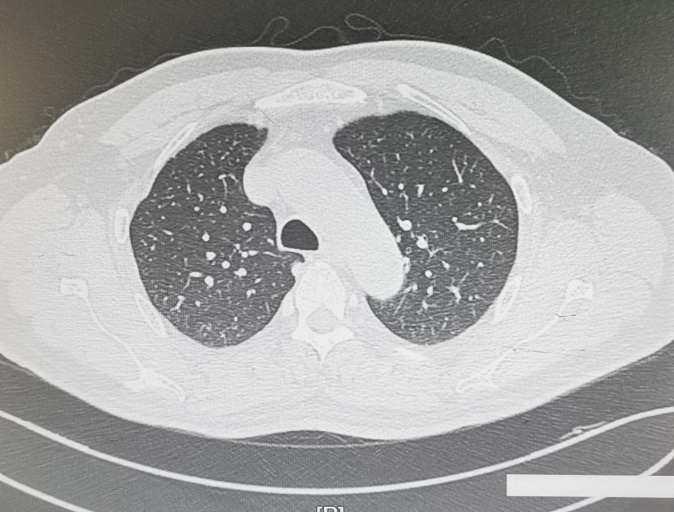

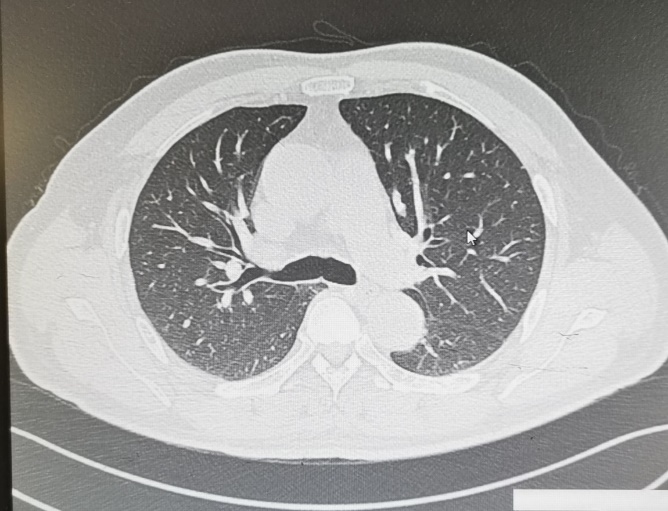

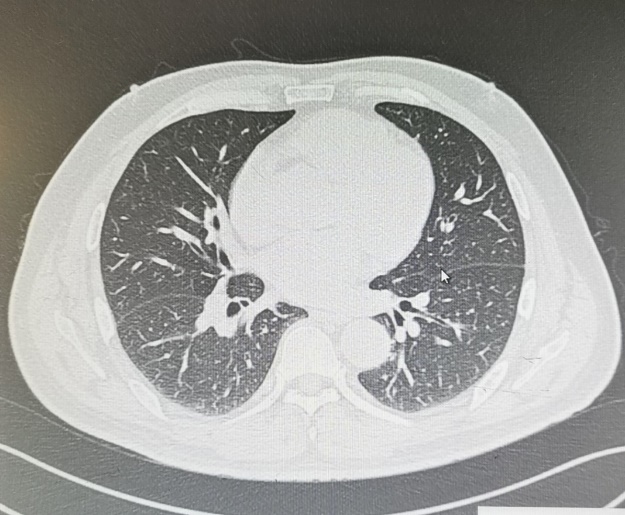


**Controls NO6：**Total score 0 =up: 0(R)+0(L) **+** middle:0 (R)+0(L) **+** low :0(R)+0(L)


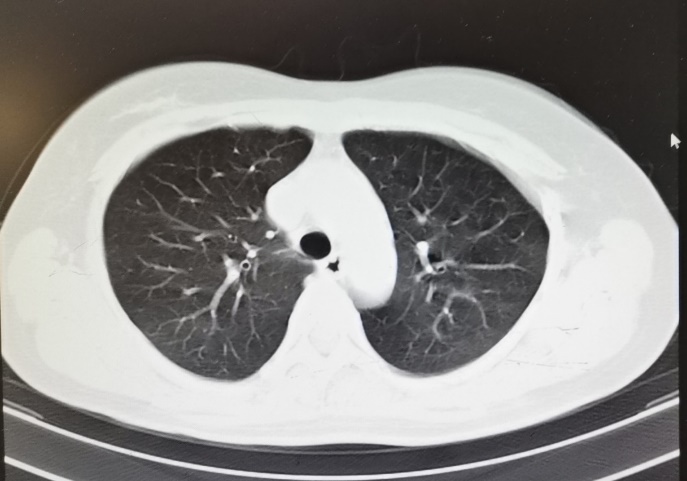

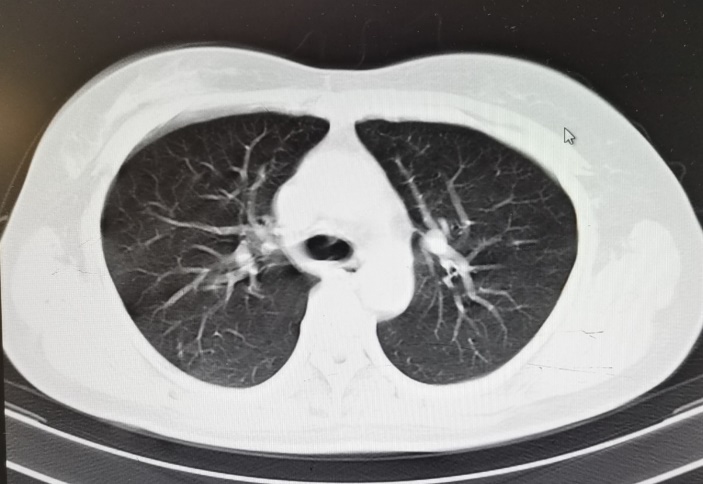

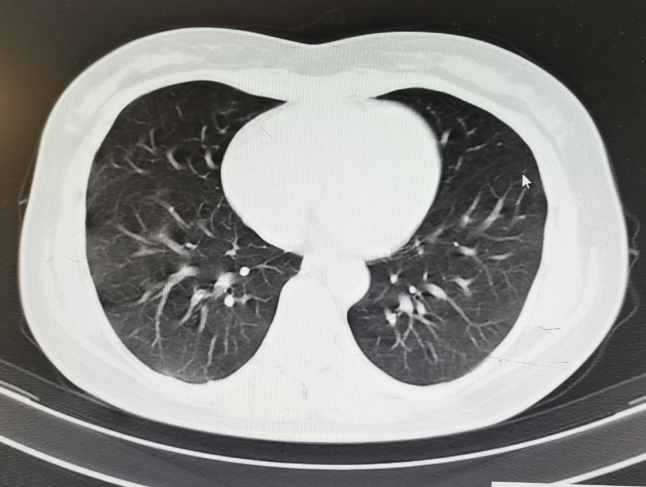


**Controls NO7：**Total score 0=up: 0(R)+0(L) **+** middle:0 (R)+0(L) **+** low :0(R)+0(L)


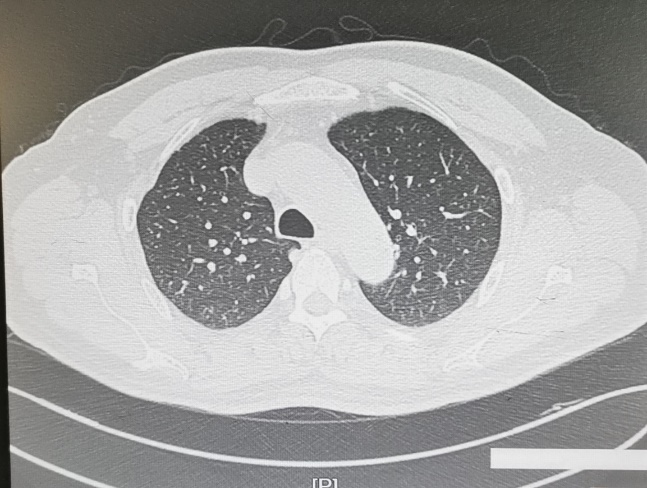

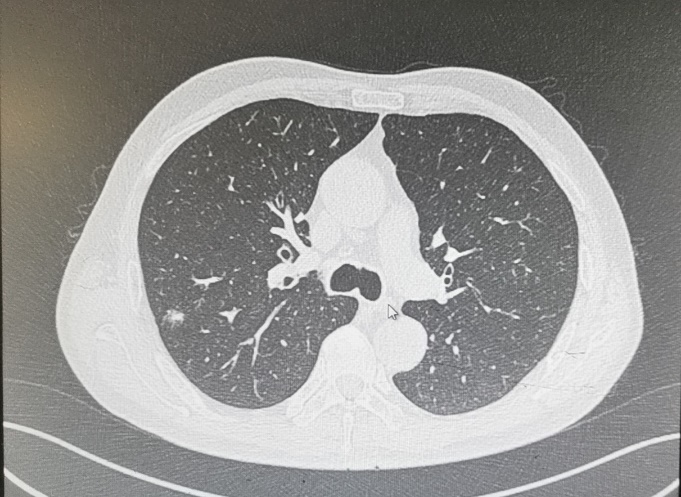

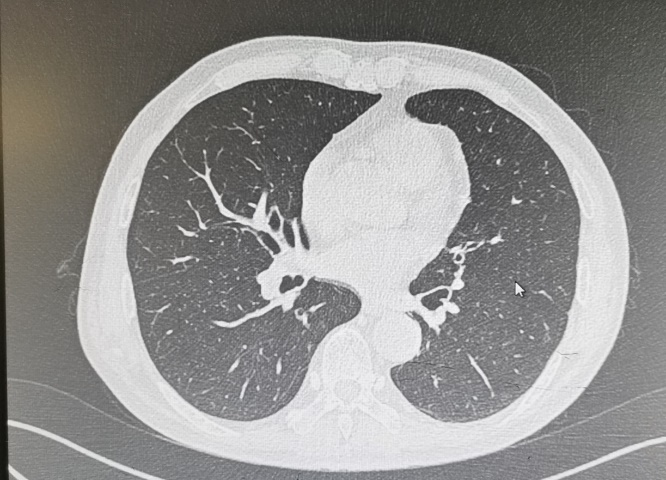


**Controls NO8：**Total score 0 =up: 0(R)+0(L) **+** middle:0 (R)+0(L) **+** low :0(R)+0(L)


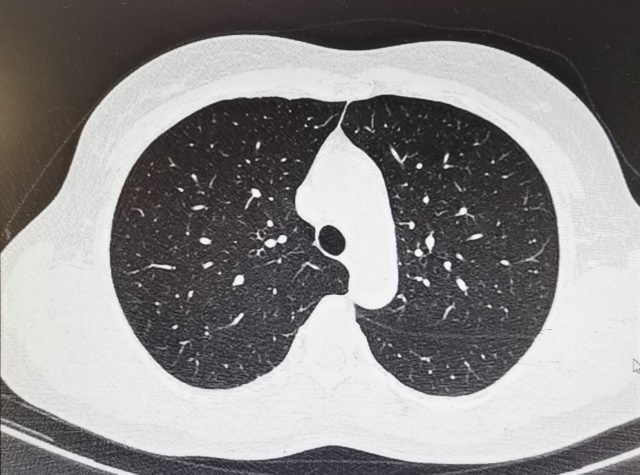

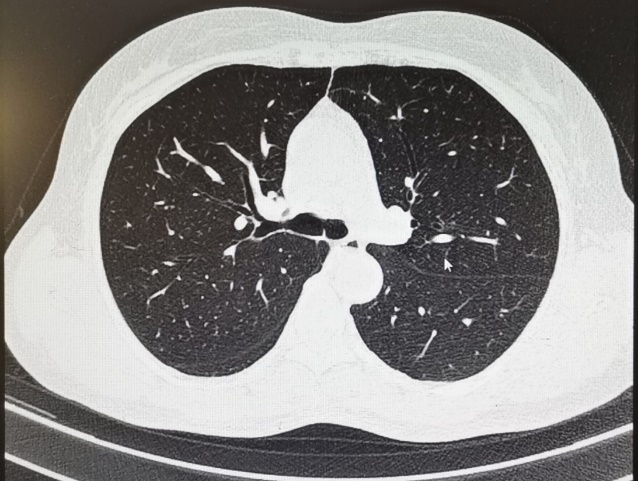

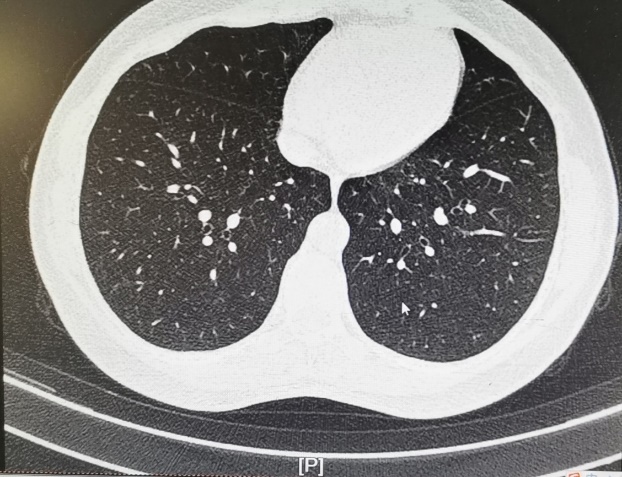

Supplement: Supplementary File 1 [file aging-13-202432-s002.docx]
